# Signaling and meaning in organizational analytics: coping with Goodhart’s Law in an era of digitization and datafication

**DOI:** 10.1093/jcmc/zmad023

**Published:** 2023-06-29

**Authors:** Jeffrey W Treem, William C Barley, Matthew S Weber, Joshua B Barbour

**Affiliations:** Department of Communication Studies, The University of Texas at Austin, Austin, TX, USA; Department of Communication, University of Illinois Urbana–Champaign, Champaign, IL, USA; Department of Communication, Rutgers University, New Brunswick, NJ, USA; Department of Communication Studies, The University of Texas at Austin, Austin, TX, USA

**Keywords:** analytics, data, signaling, metrics, measurement, commensuration

## Abstract

The future of work will be measured. The increasing and widespread adoption of analytics, the use of digital inputs and outputs to inform organizational decision making, makes the communication of data central to organizing. This article applies and extends signaling theory to provide a framework for the study of analytics as communication. We report three cases that offer examples of *dubious*, *selective*, and *ambiguous signaling* in the activities of workers seeking to shape the meaning of data within the practice of analytics. The analysis casts the future of work as a game of strategic moves between organizations, seeking to measure behaviors and quantify the performance of work, and workers, altering their behavioral signaling to meet situated goals. The framework developed offers a guide for future examinations of the asymmetric relationship between management and workers as organizations adopt metrics to monitor and evaluate work.

Goodhart’s Law states that “When a measure becomes a target, it ceases to be a good measure” (Strathern, 1997). This axiom captures the idea that data gathering and analytics can be subverted, distorted, or flawed as individuals and organizations adjust behaviors to meet situated goals. This idea’s relevance has increased alongside growing reliance on analytics to make sense of organizational problems, guide decision making, and assess worker performance (Beer, 2016; Berman, 2018; O’Neil, 2016). We position the practices of *analytics*, defined as the use of digital inputs and outputs to inform organizational decision making, as communicative phenomena in which the representation of computation mediates the performativity of individuals’ actions (Barbour et al., 2018). This perspective emphasizes that communication always accompanies the use of analytics. Communication creates data, facilitates decision making regarding how data are processed, organizes data in distinct ways, and distributes outputs to stakeholders. This article explains why these practices matter and how the activities involved in analytics work make Goodhart’s Law inevitable.

Decades of research on computer-mediated communication (CMC) use within organizational contexts positions workers as active in shaping the use of communication technologies to accomplish situated goals (e.g., Orlikowski, 1992; Zammuto et al., 2007). This literature argues that technologies have no singular, predetermined purpose; instead, technologies are flexible and derive meaning in use. However, more recent scholarship discussing organizational analytics takes a more deterministic approach, seeing analytics as a means of uncovering hidden and useful insights in data, and focusing on how organizations can develop competencies related to the application of outputs to inform decision making (Chen et al., 2012; Ransbotham et al., 2015). The purported value of analytics depends on the incorporation of large quantities of data, the capture of hard-to-oversee behavioral processes, and elimination of biased individual decision making (McAfee et al., 2012). Yet, analytics always involves computation, classification, representation, or transformation, and as such, analytics practices involve myriad choices, purposeful and incidental, regarding what inputs are used and what outputs represent. Analytics are processes through which organizations *produce* rather than find meaning.

Analytics work is a human process. Workers, through their ongoing use of CMC, take actions that produce data constituting analytic inputs, and audiences evaluate associated outputs. This dynamic, where workers make decisions about behavioral activities and organizations decide how to best analyze that activity, indicates the need to center the communicative constitution and contested enactment of analytics. Doing so recognizes a future of work where management uses CMC to track, monitor, surveil, and evaluate the efficiency and effectiveness of employees (e.g., Beer, 2016; Flyverbom, 2019) and workers adjust uses of CMC in response (e.g., Hanusch, 2017; Kellogg et al., 2020; Maiers, 2017).

The intersection of two facets of CMC in organizational life will shape this future: (a) the abundance of information regarding worker activities and (b) the development of methods to construct meaning from that information, detailed as follows. The dramatic increase in availability of information about workers and work connects directly to a set of processes linked to the ubiquity of CMC in contemporary organizational life: datafication (Christin, 2020; Hansen, 2015; Van Dijck, 2014), digitalization (Rahrovani, 2020; Timonen & Vuori, 2018), and quantification (Berman, 2018; Mazmanian & Beckman, 2018; Mennicken & Espeland, 2019; Moore & Robinson, 2016). Collectively and individually these processes contribute to the large-scale production and capture of human activity through digital records. A result of these changes is greater behavioral visibility in organizations, where activities, information, and interaction once isolated or ephemeral are now accessible to managers and workers (Leonardi & Treem, 2020).

Efforts by management to use communication technologies to direct, evaluate, and control workers’ behaviors are not new (e.g., Beniger, 1986; Yates, 1993; Zuboff, 1988). Scholars have documented how the classification of data related to technology use (Bowker & Star, 2000) and the visibility of communication and work (Star & Strauss, 1999; Suchman, 1987) have implications for the evaluation of workers and workers’ choices regarding technology use. This body of research reveals persistent tensions among organizational efforts to increase labor value, workers’ desire to assert agency over their behaviors, and the mediating role of communication (Burawoy, 1979; Mazmanian et al., 2013; Porter & van den Hooff, 2020). However, the use of CMC to facilitate contemporary applications of organizational analytics differs from historical contexts in a couple of meaningful ways.

First, the past few decades have seen the growth of computer-supported cooperative work (CSCW) as an area of distinct study (Greif, 1988; Grudin, 1994), and the development and widespread adoption of CSCW tools in organizations that allow for more distributed, fluid, and decentralized forms of work (Ackerman, 2000; Grudin & Poltrock, 2012). A central focus of this work is how using communication technologies alters users’ experiences of visibility or awareness and the consequences this visibility or awareness might have for individuals’ behavior and evaluations of others (Dourish & Bellotti, 1992; Erickson & Kellogg, 2000). Moreover, growth in internet connectivity, online software applications, and more affordable cloud-based storage means individuals are less reliant on employers to support communication tools. As a result, most workers have a plethora of options in how, when, and where they communicate. The massive volume of CMC use in modern organizations generates data for potential analysis of the specific actions taken by workers and the content of communicative exchanges. Organizations face a paradox resulting from workers’ broad accessibility and use of CMC: They potentially have access to vast amounts of data regarding the behaviors of workers, but the meaning of these behaviors is simultaneously more difficult to discern.

Second, the past two decades have seen global growth in what has been termed platform work, jobs managed through workers’ use of, and interaction with, digital platforms (Hoang et al., 2020; Howcroft, & Bergvall-Kåreborn, 2019). Examples of platform work include gig work such as ride-sharing (Uber, Lyft), food delivery (DoorDash, Deliverroo), or online piecework (Amazon’s MTurk). These work environments enact decisions regarding job assignments, compensation, and evaluation through analytic tools often guided by algorithms. The platforms mediate the communication between workers and organizations (Bucher et al. 2021; Gruszka & Böhm, 2022). Although the individual’s labor is embodied through the physical actions they take, all that is visible and communicated to the organization are data representing that labor (Mateescu & Ticona, 2020). Unlike previous organizations anchored materially in physical headquarters, job sites, or retail spaces where work is observable, in modern organizations, work is increasingly *seen* through data representing activities.

As organizations have encountered this growing wealth of data, they have increased efforts to structure, measure, and evaluate social phenomena to produce interpretable and actionable outputs (Berman, 2018; Espeland & Stevens, 1998; Hansen, 2015; Petre, 2021). This trend has been well-documented in work contexts (Beer, 2016; Mazmanian & Beckman, 2018; Ranganathan & Benson, 2020). These regimes of measurement are commonly associated with processes of commensuration, which is defined as “the comparison of different entities according to a common metric” (Espeland & Stevens, 1998, p. 313). Commensuration allows organizations to develop rankings, scores, or other forms of evaluation that ascribe comparative value to measures and tie that value to the work of individuals, teams, and organizations. When outputs of measurement systems are used for evaluation or rankings they create forms of valuation in which actors can make claims that certain individuals, organizations, or ways of behaving should be favored over others (Beer, 2016). Processes of commensuration then shape resource allocation, including attention, financial investment, and increased status such that those rated higher in commensurate metrics are likely to retain resources and keep their higher ratings in a reinforcing cycle (Sauder & Espeland, 2009). These processes of commensuration exemplify the centrality of power dynamics in the practice of CMC-facilitated analytics work even while logics of neutrality and rationality ascribed to measurement efforts tend to obscure the importance of power in analytics (Hansen & Flyverbom, 2015; Moore & Robinson, 2016; Uldam, 2016).

To navigate a future of work rife with analytics, we need a lens to understand and study the practice of analytics. We argue that the practice of analytics is fundamentally communicative in at least two important ways. First, organizations use analytics to make meanings from assemblages of data. Second, analytics are communicative in the sense that workers’ behaviors provide signals interpreted in various ways in the form of data use. Analytics mediate dynamics of meaning making in organizations, and organizations and individuals will engage in data signaling and interpretation in efforts to meet situated goals.

## Data as signaling in analytics work

Signaling theory (for review, see Connelly et al., 2011) provides an especially useful framework to examine how the practice of analytics operates to derive meaning and insights from data in the workplace. Signaling theory developed in parallel in economics (Spence, 1973) and biology (Maynard Smith & Harper, 2003; Zahavi, 1977) to explain how individuals evaluate the abilities of others based on limited or incomplete information. Signaling theory holds that we often make decisions in contexts of uncertain, incomplete, or asymmetrical information, and individuals aware of this dynamic seek to derive or provide information that will increase the likelihood of advantageous outcomes. Signals produced by humans are not neutral, but rather, they are intended to communicate a specific belief, ability, or intention (Donath, 2007).

Several principles of signaling theory are relevant to the use of CMC and the growth of analytics in organizational settings. First, the theory recognizes the difficulty in assessing the honesty and reliability of signals. That is, incentives exist to produce signals that others will evaluate favorably and observers will seek more accurate means of assessing signals (Maynard Smith & Harper, 2003). In essence, we communicate signals in an effort to shape or control the assessments and associated behaviors of particular audiences. However, signals differ in their usefulness, and those evaluating signals may differ in their ability to interpret them. In organizational contexts, data associated with a worker’s activities operate as a signal, and analytics practices consist of efforts to evaluate that signal in a manner that is useful. Given the uncertainty that can accompany signaling, perceived cost often operates as a proxy for evaluating abilities and attributes (i.e., owning fancy cars is associated with wealth; being a board-certified physician is associated with medical expertise). However, as Donath (2007) noted, the low cost of communication production, dissemination, and modification online can exacerbate the difficulty of evaluating signals in CMC. Indeed, a number of scholars have examined how CMC facilitates many forms of deception (for review, see Hancock, 2007).

Second, another principle of signaling theory posits that assessing the value of signals is more difficult in contexts with more signaling and where the information available changes frequently (Connelly et al. 2011; Steigenberger & Wilhelm, 2018). In organizational environments where CMC are constantly producing more data, the sheer volume of signaling can be overwhelming. For example, Feldman and March (1981) noted that organizations often seek and gather far more information than needed when making decisions because this practice signals thoughtfulness and rigor. Evaluating signals in a signal-rich context is a difficult endeavor.

Guided by these principles, signaling theory explains Goodhart’s Law in this way: Measures become ineffective when based on the faulty assumption that the signals they incorporate are honest, reliable, static, or interpreted accurately. In practice, measures can become decoupled over time from the organizational activities they mean to capture and evaluate. Drawing on signaling theory, the future of work can be seen as a strategic, iterative cat-and-mouse game between organizations seeking to quantify the performance of work and workers altering their behavioral signaling (in ways that call into question the validity and reliability of measures).

To extend this theory as a framework for understanding the role of signaling in contemporary organizational analytics practices, we present three case studies based on ethnographic work conducted by the authors. Although these cases are part of broader research projects involving interviews, observations, and document analysis (Barley, 2015; Jensen et al., 2022; Weber & Treem, 2016), we confine our reporting to brief vignettes describing instances of analytics work. These empirical narratives show (a) the challenges that emerge as workers seek to address situated needs associated with the use of digital metrics as evaluative tools and (b) the forms of mediation that result from the practice of analytics. In a technology sales organization, management implemented a scoring system to evaluate employees’ uses of social media, and workers exhibited *dubious signaling* in an attempt to game the system. In an organization conducting research on atmospheric science, members exhibited *selective signaling* by omitting information from digital presentations of data. And analysts in healthcare research, aiming to establish their value for the larger home organization, exhibited *ambiguous signaling* by using metrics read differently by different stakeholders to their detriment. We conclude by discussing how this signaling framework can help scholars study analytics in work contexts.

## Case 1: Dubious signaling

**We define dubious signaling as occurring when workers engage in behaviors with the goal of producing misleading, false, or questionable data**. In dubious signaling, the form of measurement and evaluation does not reflect the forms of behavior or activity it means to capture. When the data used in measures become decoupled from the underlying behavior the organization seeks to track, the validity of the measures is untrustworthy. Because employees often have explicit incentives to meet organizational targets, they may engage in signaling activities even if the signaling has little or no connection to broader outcomes the organizations seek. Dubious signaling may be especially common as workers try to game a system to hit targets.

### Beta Corp. and the implementation of TechMetric

This case uses data based on ethnographic study of work at Beta Corp. (pseudonym) conducted at regular intervals between February 2014 and September 2017. Observations of team activity and interviews with workers on those respective teams took place for 4 months during the study duration. Beta Corp., an international technology sales organization (100,000 employees globally; 3,000 sales employees) underwent a digital transformation that began in the middle of 2014 and concluded approximately 2 years later in 2016. During this time, the company shifted from an *ad hoc* measurement system that examined the daily activities of sales workers based on the specific requests and desires of individual managers to a unified, organization-wide system for tracking worker behavior. The core of this transformation was the introduction of TechMetric, a digital measure developed to capture worker behavior and interactions on a suite of online digital platforms including LinkedIn, a custom client communication portal, an enterprise social networking platform, Google+, Twitter, and other regionally specific social networking sites. Beta Corp. management’s beliefs that an increase in digital activity would help establish and deepen relationships with prospective clients, and, in turn, would contribute to increased sales drove the investment in and implementation of TechMetric.

In practice, TechMetric provided a score for each employee that was calculated by an algorithm applying an opaque aggregation of desired behaviors such as posting updates to the enterprise social networking site, updating information on LinkedIn, and entering data into the online sales tracking system. Scores ranged from 0 to 100 and were recalculated on a weekly basis to capture changes in employee behavior. To drive engagement with the metric, Beta Corp. integrated TechMetric scores into employee performance plans, and employees set goals for their scores. To be promoted to the next level within the organization, employees had to attain a minimum score of 35. The rollout of TechMetric involved extensive training communicating the types of online behaviors that would be reflected in the scoring. Each sales team completed a 1-hr group training about digital sales practices and an additional 2–5 hr of online training depending on the job role. Managers had to complete additional training.

Following the implementation of the new system, TechMetric operated in the background of day-to-day tasks, but was nevertheless an important component of overall work within the organization. Shortly after implementation, employees expressed frustration with the opacity of TechMetric and the lack of clear connections to sales outcomes. Seamus, an information technology sales representative on the Northern Europe team explained that he had adapted his day-to-day work routines to achieve the required TechMetric score, but he struggled because activity such as posting product information to Twitter rarely generated new sales leads. He commented:

I do what I have to do [to meet my TechMetric goals]. I try to be a team player so that I don’t get red stats [low scores], but at the same time, I want to close deals so that it drives my revenue, which is the main KPI [key performance indicator], and that’s where my time is really supposed to be.

Regardless of what the metric actually measured or what behavior it influenced, sales representatives such as Seamus wanted to avoid “red stats” and associated reprimands. Seamus went on to explain that he and his teammates were often confused about what behaviors influenced the TechMetric score, and Beta Corp. did not provide a clear accounting of the calculation of the score. Seamus and his team used the internal messaging system to try to share their hunches about the behaviors influencing the score, and he noted, “If there’s any success stories, they would be shared by whoever it was, with details about how they went about it and what they did, on the social or business side.” Workers searched for activities that resulted in a higher TechMetric score. The opacity of the metric and the disconnection between the numerical scores and employees’ sales goals prompted questions from employees about its utility. Yet, despite this ambiguity around TechMetric, managers felt an obligation to encourage employees to act in ways that would increase the metric.

Several months after the implementation of TechMetric, we observed workers engaging in behaviors meant only to boost their scores and not at all related to sales activities. For instance, to increase overall scores, sales workers had friends engage with online profiles by creating fake email accounts and registering on sales workers’ social pages. Another representative had family and friends from her home in Spain visit her external-facing sales webpage to boost her page metrics. Her family members would use the “Chat with a Sales Representative” tool to boost the “inbound contacts” numbers, which improved her TechMetric score. On the Northern Europe team, a sales representative discovered he could boost his score by visiting his webpage from his mobile phone. He shared this “hack” with colleagues and many other sales representatives replicated it. This dubious signaling further undermined the value of TechMetric as a reflection of workers’ digital sales activities. For instance, the TechMetric program sought to encourage more personalized use of Twitter and LinkedIn. Yet, representatives explained that teammates commonly shared tweets and LinkedIn posts copied from other sales representatives. The measured behavior became a record of copying and pasting activity, not personalized engagement or the creation of meaningful content.

As the preceding demonstrates, the goal of measurement from the workers’ perspective was to achieve the best possible score. Employees risked promotions and faced remedial training if their scores were too low, but a higher score had little tangible value aside from avoiding scrutiny and reprimands. Sales outcomes, not TechMetric, funded salaries and annual bonuses, which magnified the problem of the lack of connection between the process and outcome. That misalignment also undermined motivations to correct or specify the dubious signal. Instead, TechMetric fostered gaming the data and producing dubious signals. The analytics practices at Beta Corp. meant to capture if workers produced the signals that resulted in improved numbers, and therefore, workers focused on the activities that produced those signals.

The case of Beta Corp. and TechMetric demonstrates two important aspects of dubious signaling in the context of CMC. First, the fact that workers and management both focused on the value of the metric, seeking increases in the scores, created an insidious cycle that rewarded dubious signaling and obscured it from scrutiny. When workers covertly gamed TechMetric and their scores rose, they were likely to be praised and encouraged. Indeed, we observed managers telling workers with lower scores to seek advice from team members engaging heavily in dubious signaling. Second, even if workers did not wish to engage in dubious signaling, measurement was relative. So, workers risked being disadvantaged by acting as intended by TechMetric. The effectiveness of dubious signaling created normative and functional pressures for others to act to increase TechMetric scores. Because management focused on the numeric representation of the measure, and not the underlying behavior change, the dubious signals further decoupled any links between work processes, analytics, and outcomes.

## Case 2: Selective signaling

**We define selective signaling as workers’ choices to omit, hide, or withhold signals of behaviors and activities to prevent that information from being used in measurement or evaluation**. Workers often have insight into the forms of data and information used in decision-making processes. By selecting and constraining the information used in organizational analytics, workers shape what it communicates to decision makers. Selective signaling communicates that workers are acting cooperatively and obscures active efforts to conceal information or influence meaning making.

### National Center for Atmospheric Research and sharing data with the Range Operations Command

This case uses data from observations of work at the National Center for Atmospheric Research (NCAR) conducted between September 2011 and August 2012. NCAR’s “Range Team” developed numerical weather prediction (NWP) systems to support operations at military test ranges across the United States. The Range Team accomplished this work through a multi-year sponsorship from the “Range Operations Command” (ROC). The Range Team used cutting edge scientific knowledge to develop analytic tools to forecast weather at test ranges in multiple, different regions from sub-zero tundra to urban areas where the military tested equipment, including explosives, vehicles, and missiles.

Each range required unique, customized analytics. The analytics also measured the efficacy of the scientists’ ability to simulate range conditions. The scientists welcomed this complexity as a challenge and opportunity to extend their scientific knowledge, but the scientists’ research goals did not always align with the ROC’s goals of minimizing costs and maintaining effective operations. Accomplishing the Range Team’s scientific goals often required that the scientists convince the ROC to support their desired direction for work. These efforts occurred most visibly at bi-annual program reviews where researchers and ROC personnel gathered to share metrics related to NCAR and to decide which future actions the ROC would support. As the scientists developed their tools they conducted a variety of statistical analyses, each using various data as inputs and producing different data as outputs. The scientists then had to decide what about these processes they would share or withhold in interactions with ROC. Their choices of which metrics to share with the ROC embodied what we call selective signaling.

NCAR researchers recognized that program reviews were important occasions to cultivate support for their research. They spent the weeks leading into the meeting strategizing how to present. On one such occasion, Darren, the Team’s lead scientist, gathered with Neil, the lead software engineer, and Berto, an atmospheric scientist, to discuss a presentation Berto would make at the upcoming review. Darren explained their desire to communicate the value of a new technology that was showing promise in improving predictions and commented, “That’s what we’re here to strategize on: we want Erik [the director of the ROC] to get the right message.” Discussion revolved around how they could present metrics about Berto’s technique to encourage ROC’s support for this work. Their strategizing involved selecting and transforming the metrics Berto would present in his slides. Berto eventually arrived at a slide displaying several plots directly comparing the Range Team’s current analytic technique with Berto’s new technique, designed to show the superiority of the new measure. Although Berto’s explanation of the graphic suggested that his new technique predicted low-altitude wind speeds much more accurately than the team’s current technique, it used uncalibrated forecasts that did not adjust for known issues with the current analytics approach. Darren explained that using uncalibrated data would exaggerate the difference between the two techniques on the performance metric. Although this might effectively communicate the relative advantage of the new technique, it involved presenting data they knew to be misleading and therefore was not an option.

Instead, Darren proposed omitting the metric comparison altogether from the plot they presented. He explained: “But it’s dangerous … Because one of the major messages of this slide is, ‘the [current technique] that you’ve been spending all this money on isn’t reliable.’ And I don’t think that’s a message we want to send right now.” Berto agreed with Darren and mentioned that he was glad they had met to rehearse. Darren’s explanation is telling. He perceived a “danger” associated with showing these metrics. That danger was communicative: the potential for signaling that would harm the Team’s research plans. Berto agreed and deleted the plots.

The program review occurred the following day. The strategy to select favorable metrics seemed to work from the start of Berto’s presentation to ROC stakeholders. Darren described Berto’s contribution as a supplement to, not a replacement for, the existing forecasting system. As Berto received Darren’s hand-off, he stressed that the metrics he would be showing were preliminary in nature. These themes carried throughout the remainder of the presentation. Berto showed plots of the technique’s performance with data from one of the test ranges, omitting as planned the direct comparison between his technique and the system currently deployed at the ranges. Berto’s delivery acknowledged the lack of comparison explicitly: “So here you only see the results for [the new technique]. What we’re going to do in the future is compare [this technique] with the [existing technique].” Berto highlighted this lack of comparison as an indicator of the incomplete nature of the current work. Berto then moved on to show a plot comparing his new metric to data from a forecast system operated by a different NCAR collaborator. These plots demonstrated how Berto’s technique had radically improved the system’s accuracy. Berto concluded by arguing that if his technique could produce similar results for ROC, it would benefit system accuracy and require fewer computational resources.

In the discussion that followed, Lance, a senior ROC forecaster and advisor to Erik, spoke up wondering why they were showing a technique not used by the ROC. He asked if they had an actual test case that would show this technique was better than the existing model. Lance had interpreted Berto’s metrics in exactly the manner the researchers’ strategy meant to avoid. He saw Berto’s technique as a potential replacement. Darren interjected to defend Berto and assured Lance that this was not intended as a replacement to the current model. He argued, “There are reasons to believe that some combined approach may be able to draw on strengths of both approaches. So, we would resist making any decision like that because we don’t think we’re even close to being able to do that.” Neil agreed, saying, “And we think it deserves some support to see where it goes. But it doesn’t call for re-thinking the strategy here.” The scientists seemed unsure how the presentation was being received, and the room felt full of tension.

After a few more minutes of discussion, Erik, the director of the ROC, who had remained silent to this point, stated that he thought they were not yet in a position to commit to this new approach, but that it merited future consideration. He added, “Depending on how well the research works out. So we’re not being fraudulent about having this new system if we decide that it’s best to incorporate more and more of this approach, versus the traditional approach that we’ve been using.” Erik’s comment moved toward resolution. The team proceeded with their research on the new method without abandoning their existing approach.

Although the researchers took measures to shape the unfolding of program review through selective signaling, they had only partial success. The very interpretation that they hoped to avoid surfaced when Lance asked his question about Berto’s data. Following this moment, the team had to communicate to negotiate how they would proceed.

Rational models of organizational decision making assume that actors should make all relevant information available to decision makers to produce optimal decisions. As a result, organizations hold as an ideal analytics work that incorporates all relevant data and analyzes them using the most reliable and valid methods. Following this ideal, the central challenge for organizations should be surfacing, sharing, transferring, and translating knowledge; restricting information access would undermine that effort. However, this case demonstrates that in digital workplaces the challenge may not be the paucity of information, but rather the difficulty of how to sort, curate, and process the plethora of data available. Selective signaling is one way that workers manage this complexity to achieve their own ends.

The interactions at NCAR show that selective signaling can take place around communication choices regarding uses of data, applications of analytical procedures, or the sharing of results. In the case of scientists working with ROC, the researchers took steps to shape future interactions via the selective presentation and framing of data representations. In the deluge of data available in organizations, selective signaling may be less likely to be noticed. Analysts share results, present models, and produce scores, but selective signaling means that other decision makers may be unaware of alternative possibilities. Selective signaling may afford workers a communicative form of authority by shaping which data are interpreted as meaningful in analytics work.

## Case 3: Ambiguous signaling

**We define ambiguous signaling as occurring when producers and audiences have differing but plausible interpretations of the meaning and value of data presentations or analytics procedures**. A purported benefit of metrics is that they facilitate standardization and comparison across contexts. However, this value depends on the assumption that those using a metric assess its outputs in similar ways (i.e., a high number or ranking is seen by all parties as a good thing). When they do not agree, such as when they do not find data outputs valuable or do not understand their significance, it risks undermining the usefulness of organizational analytics. Unlike selective and dubious signaling, which are presented as potentially empowering to workers as a means of asserting agency, ambiguous signaling can constitute a failure of workers to communicate in a manner that meets their goals unless that ambiguity is useful to them.

### The Analytics Center and measuring requests for data

This case uses data based on observations of work at “the Analytics Center” (pseudonym), a unit that provided data to medical researchers who worked throughout a larger healthcare organization, HIRO. Initial data collection at HIRO began in 2018 and observations and field interviews focused specifically on the Analytics Center started in the summer of 2019 through the end of the year, amounting to 6 months of fieldwork. The Center facilitated access to massive data sources through relationships with clinical partners and technology firms, data-sharing, and interorganizational collaborations. They helped their clients navigate regulatory requirements, technical tasks, and intra- and interorganizational relationships to get and analyze large datasets.

The Analytics Center sought to measure the success of their work using requests-fulfilled (RF) metrics. They generated RF metrics using the Request System, a portal for receiving, managing, and monitoring requests for data from the organization. They also built an RF metrics dashboard that used the data from the Request System to communicate their metrics to the rest of the organization. They built RF metrics to capture the Center’s increasing speed in processing demands and to communicate the number of requests addressed. The RF metric served as the data output the Analytics Center chose to represent to senior leadership the value of the work being conducted. These metrics mattered because HIRO was in the midst of an ongoing budget review. HIRO was a new organization that had increased in size 100-fold in fewer than 5 years. Budgeting meant aligning their hiring, structure, and other resources with emerging priorities.

From the start, individuals at the Center recognized that measuring their work was difficult. Their function was specialized, which meant most decision makers did not have a clear sense of it. It was also highly technical. They received and processed requests for data, but that included a range of activities. As the Director of the Center explained in the meeting, “[a metric like] ‘4 requests per week’ doesn’t really communicate what we do.” Few researchers understood exactly what data they needed or even the data available, let alone the regulatory, technical, and organizational challenges involved in accessing even the organization’s own data. In early conversations about RF metrics, the Director expressed confidence, observing, “The good news is that there’s very little doubt in anyone’s mind about the importance of data. Everyone agrees with that. We don’t have to defend what we do. Also the team has been successful. We have concrete achievements.” He drew a contrast with conversations from the last year when “no one even knew what the [Analytics Center] was,” and “now we have direct exposure to leadership….we’re in every PowerPoint slide.” Nonetheless, they talked about the need to communicate to other leaders throughout the organization to develop the “message we have to keep building.” As the conversation shifted to specific projects, another member of the team took a deep breath, sighed, and said that the good news was that “we control the most important resource in this world: Data and information,” but by December of the same year, the Analytics Group would be disbanded.

As they tried to make the case for their work, the Analytics Center worked on refining the Request System and the RF metrics. Prioritizing and estimating how much time a request should and would take was difficult. They tried to distinguish between “really complex ones where it just takes weeks to even know the request” and a request for a specific, well understood dataset. They needed to communicate how they spent their time on requests. From an “accountability perspective,” they needed to document “what time is spent in data requests, coordination, skill development” to say at the end of the year that “our team has spent 100 hours in the last year on skill development.” Because of this emphasis on completion time, they designed the dashboard to represent each request’s current stage of completion and how long a request spent at each stage.

A consequence of the Analytics Center choosing to communicate about RF metrics and completion time as an indicator of work meant that those metrics were the primary information available to the broader organization about the nature of the group’s work. Those outside the Analytics Center could see indications about the increased amount of work at the Analytics Center, but had little information about the nature of that work, its contribution for whom, and to what benefit. The dashboard signaled the Analytics Center’s involvement in projects, but only the direct recipients of the Center’s services could know the value of this increasing workload.

This emphasis on completion time created a recurring problem: The dashboard displayed multiple requests that persisted despite the Center’s concerted efforts to complete them. This problem happened because of factors outside their control such as slow-to-respond partners and because of the complexity of requests. Discussing the early versions of the Dashboard, they highlighted a request and explained that the project persisted because “we call it a data request, but really it’s a project.” Requests could be straightforward deliveries, but others involved additional real-time collaboration with requesters to understand, query, clean, and assemble their data. The system offered no clear way to demonstrate this distinction. They talked about rating their sense of the difficulty or complexity of each request, but that detail did not make it into the dashboard.

Delays prompted conversations about requests that took too long. Team members discussed reassigning or deleting requests if they were stuck. Other conversations focused on projects where the requester had not responded to clarifying questions. Again, a team member remarked, “If they don’t respond, we should just delete it because it’s pulling our numbers down.” Workers expressed frustration that they were not able to effectively signal the factors delaying progress on projects. The wider organization had little understanding of these nuances associated with the RF metrics and the nature of requests. For example, in a meeting to discuss a future project, a Center collaborator explained, “we can’t write a proposal yet because we don’t know what data are really available,” indicating that she did not think the Analytics Center served this function, even though they saw helping provide a sense of available data as a core strength. After another meeting, a Center team member mentioned that her clients in the organization expressed surprise when they learned members of her team dealt with dozens of requests at any given time. The client estimated that the Center staff each had only one or two in process.

Once completed, the RF metrics dashboard could count the delivered requests, break out counts by weeks in stages, by team member, by time periods like year-to-date, and by quarter. The metrics focused on how fast they responded. The metrics missed the connection to the larger organization’s strategic priorities. The dashboard and system offered no information about how fulfilling a request contributed to the funds raised by the organization, which they often did. The metrics did not distinguish between requests that involved a simple request for data or a complex one that involved clarifying and renegotiating the nature of the request with the researcher.

Months later, the meeting after the budget decisions had been made was grim. The Center would be broken up. It was unclear to leadership if and how the Center generated enough value for the entire organization to justify its large personnel costs. Leaders also saw the Center as serving just one unit when in reality they had clients throughout HIRO. They moved Center team members into an information technology unit under different leadership, and the Center’s leaders shifted focus to supporting the transition and then returning to their other work. During the meeting, a recent hire said of his RF metrics, “I show up as zero on everything. If they were to ask me what I bring, it doesn’t look like I do anything.” He recognized that for those outside his group, the RF metric communicated the value he offered to the organization.

They meant the RF metric to convey the value of the Analytics Center’s labor, but it contributed to their disbanding because of its ambiguous signaling. Leaders outside of the Center came to see RF metrics as indicating the group was slow and overwhelmed, whereas Center staff saw the metrics as indicating they were getting faster and being well utilized, notwithstanding requests that they discounted because of their complexities. At the same time, the focus on RF metrics obscured the Center’s contributions to other outcomes that mattered to the organization such as how their work advanced the work of HIRO and their success securing funding. The Center’s belief in the value of RF metrics and the invisibility of alternative interpretations meant their metrics failed.

The ambiguous signaling in this case is the function of two different aspects of visibility: the prominence of a measure, the RF metric, that was not easily interpreted by the broader organization and the invisibility of the actual work of the Analytics Center, which obscured their value. This example demonstrates the difficulty of effective signaling in analytics work and the interdependence of signaling choices. The decision by the Analytics Team to focus on the RF metric and the dashboard reflected their belief that this measure would be easily understood and equally applicable across the organization. Yet, in focusing attention on this measure, and then doubling-down on refining it and communicating about it, the Center focused less on other efforts to communicate about their work. In their desire to simplify their analytics communication, they became too reliant on a signal that was not well understood.

## Discussion

An implicit corollary to the aphorism that data doesn’t speak for itself is that the data are speaking. Yet, as data about workers’ behaviors are more readily available to organizations, questions regarding who is using data to speak, what different audiences are hearing, and what data are saying are more pressing. The cases presented and the accompanying signaling framework described here demonstrate how the practice of analytics work constitutes the meaning of data and how organizational and individual choices regarding how it is communicated, viewed, and interpreted shape analytics work. Theoretically, we offer a signaling framework as a means to examine how workers actively shape analytics work through the use of CMC and different communicative strategies that they might employ with varied sophistication and success in efforts to meet situated goals.

Each case demonstrates the interdependent choices made by workers and the organization regarding what data would be used in analytics work and what that data were supposed to signal. At Beta Corp., TechMetric meant to capture workers’ digital sales activity. At NCAR, the data signaled the efficacy of a new predictive model. At HIRO, the RF Metric represented the contribution of the Analytics Center. In each instance, workers’ use of CMC produced the data through online activity, model runs, and operation of a digital dashboard and constituted the signaling. In turn, these signals made visible behavior that audiences then used to judge the meaning and value of workers’ activities. See Table 1 for a summary of signaling activities across the three cases.

**Table 1.** Summary of metrics and signaling across the three cases

Case metric	What metric was designed to assess	Signaling by workers	Assessment of signals by audience	Nature of signaling
TechMetric, score reflecting digital engagement	Measured activity on digital platforms based on organizational belief this behavior related to increased sales.	Sales workforce pursued but also gamed the metric, which they saw as disconnected from their real work.	Managers who pushed workers to hit their metrics	Dubious signaling occurs when the communication provides a specious signal that differs from what the metric is intended to capture. The fake engagement meant TechMetric would not function as a good indicator of sales.
NWP system, statistical measure of predictive accuracy	Measured whether new model procedure was more accurate and effective compared with existing approach.	Researchers selected which metrics to show to make their case for their new work without undermining the existing work.	Lack of complete data led collaborators to question the wisdom of continuing with the existing model even though the other metric had not been included.	Selective signaling occurs when presenters omit metrics altogether. Omitting the metric did not prevent the conversation altogether, but the presenters were still able to shape the conversation in accounting for its omission and argue for the approach they wanted.
Job requests completed	Served as a measure reflecting the contribution of the Analytics Center to the organization.	Requests completed showed how many (complex and difficult) data requests were being fulfilled, and how long jobs took.	Communication of the metric resulted in the perception that the unit was slow in completing work.	Ambiguous signaling occurs when producers and audiences have different but plausible interpretations of a metric. Requests undermined the efficacy of the unit because it created confusion regarding the nature and value of the Center’s work, regarding the value of analytics.

Communicators’ different and conflicting motives regarding their signaling underscore the need to center the study of analytics practice on visibility management (Flyverbom, 2019). Viewing analytics work in terms of visibility management takes into consideration the active choices workers take to signal how CMC technologies facilitate and constrain their efforts. At TechMetric, the dubious signals were possible because the form of communication needed to provide the signaling, engagement on social media, was easily available and understood by the workers. Alternatively, at NCAR, the belief that the selective signaling would be successful reflected an assumption that others would not be able to understand, or be aware of, the data processing options available to the scientists, which proved false. Ironically, HIRO was the only case where the workers created and selected the metric used to evaluate work, and they failed to develop an effective signal. The mediated nature of their communication helps produce asymmetric insights into how data are produced: Individuals using outputs of analytics to make decisions often have limited visibility into the constitution of data.

The increased ubiquity of CMC in organizations creates a crowded and complex signal environment. Because communication is easy to produce and distribute, communicative signaling is abundant and accessible to everyone. The sheer volume of potential signals communicating about work, with and through CMC, may increase the likelihood of the types of signaling exhibited in these cases. Indeed, research indicates that in organizational contexts where the volume and variability of signals is high the ability to reliably evaluate signaling is likely to be reduced (Connelly et al. 2011; Steigenberger & Wilhelm, 2018). For organizations, the ease of signaling through CMC can fuel the appetite for analytics and the illusion that greater data will yield more effective decision making. However, CMC also creates more *turbulence* in communication (Litt & Hargittai, 2014) where the activities of others can alter perceptions of signals, and signals can be differentially interpreted. That turbulence can make establishing reliable measures of communication extremely difficult. The dynamism of digital communication means that Goodhart’s Law is likely to preside over the future of work.

Researchers and practitioners would be well served to recognize that even as our analytical tools become more powerful and the data used as inputs more vast, efforts at measurement are always representations that are therefore incomplete. Flyverbom (2022) described our contemporary organizational environment as “overlit” with the use of digital tools. He noted that analytics can operate as a powerful spotlight on behaviors, shedding light on activities that used to be kept in the dark, and producing a space for people to make easier assessments. But the bright light can also create shadows that obscure and divert attention, and it can attract performances from those seeking unwarranted attention. This asymmetric view of transparency is already evident in work environments, such as gig work, where management uses digital tools to monitor workers, and workers seek ways to evade, game, or subvert these systems of control (Cameron, 2022; Cameron & Rahman, 2021). Ongoing research on the transparency of digital tools like algorithms needs to consider that merely making visible the procedures used in practices of analytics does not mean that the consequences of these practices will be known or understood (Carter & Egliston, 2021).

A plausible interpretation of the cases, specifically the examples of dubious and selective signaling, would be that they are efforts of resistance against managerial policies that were viewed as ill-conceived or ineffective. Certainly, the efforts of workers to manipulate managerial evaluations of work are consistent with centuries old forms of labor resistance (e.g., Burawoy, 1979). However, the efforts of workers in these cases differ from traditional understandings of resistance in the sense that it is not necessarily the case that workers desired changes in policies or structures. As long as workers derived the desired outcome from their signaling, they offered little overt or explicit resistance. Rather, signaling is better understood as an effort of workers to exert and assert control over how they make their work visible and how others evaluate their work. This interpretation would align with research on managerial efforts to control workers’ activities and beliefs through communication and the flexibility afforded to workers through CMC as facilitating efforts to reassert control through signaling (Beniger, 1986; Yates, 1993). The cat-and-mouse game of organizational analytics is about control over meaning. Those evaluating signals such as specific data or reports of data that represent behaviors seek to develop methods of aggregating, extracting, or eliciting an accurate and meaningful reflection of activity. In turn, workers provide signals aimed at shaping the communication of activity in ways that meets situated goals. At times, these dueling efforts for control may agree, but at other times, they are likely to be contested.

These cases emphasize that future research should focus on the visibility of signaling practices among workers, especially instances where activities remain invisible, undetected, or ignored. In the instance of TechMetric, the fact that the dubious signaling resulted in higher scores, and that managers were under pressure to increase the scores of team members, meant that the actual behaviors of workers remained unexamined. In the case of selective signaling, a domain expert in the audience noticed the omission, prompting the communicators to justify their actions. However, in the case of ambiguous signaling, the inability to connect the measurement to distinct organizational benefits led to the discontinuing of the measurement efforts altogether. An important distinction is that in the case of dubious signaling and selective signaling, the relationship between the signal and the assessment in that analytical process was known by workers; workers knew how the information was likely to be used and could adapt communication strategies. In the case of ambiguous signaling, the challenge was that the connection between the measurement and the signal may not be apparent. They were eager to provide the data that would be valued, and they had a number of different options for what that data might be, but in the end, what they produced did not provide the clarity they sought.

Future research should examine how workers behave in environments where they may have limited insight into the types of measurement and evaluation applied in organizations. Examples that involve asymmetric visibility should be especially useful to investigate the functioning of ambiguous signaling (Hatuka & Toch, 2017). For example, organizations increasingly use “artificial intelligence” software to screen application materials and interviews of prospective employees. As a result, applicants may be rejected with little understanding of why or even that no human ever viewed their materials. Related to this practice, scholars have become increasingly interested in folk or lay theories that technology users adopt relative to how they are surveilled and evaluated online (DeVito, 2021; DeVito et al., 2018). This line of research indicates that even in the absence of visibility into the use of analytics for evaluation, workers will adjust communicative signaling based on their personal beliefs or group norms—their theories of how analytics work.

Finally, although the discourse around the ubiquity of CMC in the workplace tends to focus on how technology increases opportunities for monitoring, surveillance, and evaluation of workers, the empirical cases presented here indicate that CMC can empower workers. These findings concur with research on the use of CMC by social activist groups and groups opposing oppressive governments that demonstrate that the visibility of communication can threaten to expose activities and allow for subterfuge, evasion, and expression (Pearce & Vitak, 2016). Growth in working from home during the COVID-19 pandemic prompted increased surveillance by employees in the form of keyboard and monitor tracking but also led to counter-innovations by workers like cursor movers, the use of non-approved applications or software, and other behaviors designed to resist organizational actions. The growth of CMC has empowered organizations to track and discipline workers, and the digital future of work also offers workers opportunities to reassert control over their signaling through the weaknesses of dubious signaling, in making choices about selective signaling, and by capitalizing on the ambiguity in signaling.

This multicase study demonstrates that analytics are far from neutral and objective, and that organizations would be wise to consider how workers understand forms of measurement and how to communicate them. As the volume and forms of data available to organizations expand and efforts by organizations to structure and apply that data grow in frequency and complexity, questions about how communication constitutes the meaning of that data take on increasing importance. The future of work will be defined by interdependent efforts by organizations to utilize measures to increase efficiency and effectiveness from workers, and workers will seek to communicate to align with and achieve desired outcomes or confound them, which undermines measurement efforts. Heeding Goodhart’s Law means understanding the many ways datafied and digitized workplaces create new forms of visibility management and communicative signaling by workers.

## Data Availability

The data presented in this article are not publicly available and cannot be shared due to agreements to protect the privacy and confidentiality of the participants.
